# DiSNPindel: improved intra-individual SNP and InDel detection in direct amplicon sequencing of a diploid

**DOI:** 10.1186/s12859-015-0790-y

**Published:** 2015-10-24

**Authors:** Jizhong Deng, Huasheng Huang, Xiaoli Yu, Ji Jin, Weisen Lin, Fagen Li, Zhijiao Song, Mei Li, Siming Gan

**Affiliations:** 1 College of Engineering, South China Agricultural University, Wushan Road, Guangzhou, 510642 China; 2 State Key Laboratory of Tree Genetics and Breeding, Chinese Academy of Forestry, Xiangshan Road, Beijing, 100091 China; 3 Department of Computer Science, Guangdong University of Science and Technology, Xihu Road, Dongguan, 523083 China; 4 Research Institute of Tropical Forestry, Chinese Academy of Forestry, Longdong, Guangzhou, 510520 China

**Keywords:** Diploid, Insertion-deletion (InDel), Resequencing, Single nucleotide polymorphism (SNP)

## Abstract

**Background:**

Amplicon re-sequencing based on the automated Sanger method remains popular for detection of single nucleotide polymorphisms (SNPs) and insertion-deletion polymorphisms (InDels) for a spectrum of genetics applications. However, existing software tools for detecting intra-individual SNPs and InDels in direct amplicon sequencing of diploid samples are insufficient in analyzing single traces and their accuracy is still limited.

**Results:**

We developed a novel computation tool, named DiSNPindel, to improve the detection of intra-individual SNPs and InDels in direct amplicon sequencing of a diploid. Neither reference sequence nor additional sample was required. Using two real datasets, we demonstrated the usefulness of DiSNPindel in its ability to improve largely the true SNP and InDel discovery rates and reduce largely the missed and false positive rates as compared with existing detection methods.

**Conclusions:**

The software DiSNPindel presented here provides an efficient tool for intra-individual SNP and InDel detection in diploid amplicon sequencing. It will also be useful for identification of DNA variations in expressed sequence tag (EST) re-sequencing.

**Electronic supplementary material:**

The online version of this article (doi:10.1186/s12859-015-0790-y) contains supplementary material, which is available to authorized users.

## Background

Single nucleotide polymorphisms (SNPs) and insertion-deletion polymorphisms (InDels) have become the most commonly used DNA markers because they are co-dominant, abundant within the genome and amenable to flexible genotyping techniques [1, 2]. They could be derived from a number of sources, including re-sequenced polymerase chain reaction (PCR) amplicons, genomic libraries and expressed sequence tag (EST) datasets [3]. From these, although genomic and EST resources, in large scale in particular, tend to be produced with the aid of next-generation sequencing (NGS), amplicon re-sequencing based on the automated Sanger method remains popular for a spectrum of genetics applications. For instance, Sanger sequencing of PCR fragments is needed to reveal sequence variations among races and/or lines in a specific gene (e.g., *tb1* gene in *Zea mays* [4]); also, the candidate gene/region mapping strategy represents a more feasible alternative to random whole-genome SNP mapping in association studies for species with limited linkage disequilibrium (e.g., trees [5]), of which the candidate SNPs have to be generated with Sanger sequencing. In addition, Sanger sequencing is still the method of choice for DNA marker development in cases that the budget is limited and the number of markers required is not very large.

Amplicon re-sequencing can be performed via sub-cloning or direct sequencing [6]. Sub-cloning method results in single-strand sequence in each trace file (chromatogram), and SNPs and InDels are thus identified between or among traces using alignment approaches [7]. However, direct sequencing involves generally both strands (alleles) of a diploid, and double peaks will present for a single base position in case of a SNP and for nearly all positions subsequent to an InDel, which have to be distinguished using specific algorithms. As sub-cloning is time-consuming, laborious and expensive, direct sequencing has been the preferred assay. To date, several tools have been developed to detect intra-individual SNPs and InDels in direct amplicon sequencing of diploid DNA samples, including those recently developed packages Mutation Surveyor (http://www.softgenetics.com/mutationSurveyor.html), novoSNP [8] and PolyPhred [6, 9]. However, these packages are insufficient in analyzing solely single sequencing traces. For example, all the above packages require a reference sequence, which would be a constraint when no reference sequence is available, such as an intron region in EST re-sequencing. In particular, PolyPhred combines multiple individuals (e.g., ≥ 8) to guarantee essential accuracy, inhabiting its utility for single samples, such as either parent of an F_1_ or backcross population that serves in plants and animals as the common mapping pedigree and segregating markers have to be originated from the heterozygous parent(s). In addition, a more recently reported package PrimeIndel [10] can detect InDels without a reference sequence, but it needs two sequences derived from the double peaks within a certain range, which could be tedious. Moreover, the accuracy in single trace detection is still limited for the existing software tools (see Results below).

In this paper we present a novel computational tool that enables automatic detection of intra-individual SNPs and InDels in direct amplicon sequencing of a diploid sample needless of a reference sequence. Because wave noises impaired the quality of a sequence trace and were directly correlated with the false-positive and missed SNP rates [6, 8], we introduced Haar wavelet transformation [11] to decompose the wave (base) signal of a trace file at multiple-level resolution and filter out the noise of high-frequency sub-signals. The Haar wavelet approach is advantageous in simplicity, small CPU time and highly accurate and fast transformation [12] and appears very attractive in image coding, edge extraction and binary logic design [13]. Subsequently, we used simulated data to train Levenberg-Marquardt (LM) algorithm [14, 15] based back-propagation neural networks (BPNN [16, 17]) for intra-individual SNP diagnosis and also used real trace data to test the performance of the trained algorithm. BPNN is advantageous in non-linear perception, self-learning, self-adaption and generalization ability [18]. LM is a modified method for training BPNN that can improve greatly the back-propagation convergence speed and the prediction accuracy [19]. Finally, for intra-individual InDel detection, we employed a stepwise allelic base alignment algorithm to compare dynamically the primary and secondary base calls downstream of a potential InDel. We benchmarked our method, termed DiSNPindel, with other detection packages (Mutation Surveyor, novoSNP, PolyPhred and PrimeIndel) and showed that improved accuracy was achieved with two real datasets tested.

## Implementation

### Overview

DiSNPindel is implemented with the main sequential steps for SNP detection: ‘1. Open a file’, ‘2. Find SNPs’, ‘3. Manual modification (optional)’ and ‘4. Save result’, each corresponding to a button or box on the interface (Additional file 1: Figure S1). If continuous double peaks are found, the ‘Switch to Indel detection’ button can be clicked to ‘Indel detection’ interface, where ‘3. Find Indels’, ‘4. Manual modification (optional)’ and ‘5. Save result’ were designated for the specific functions (Additional file 1: Figure S2).

**Fig. 1:**
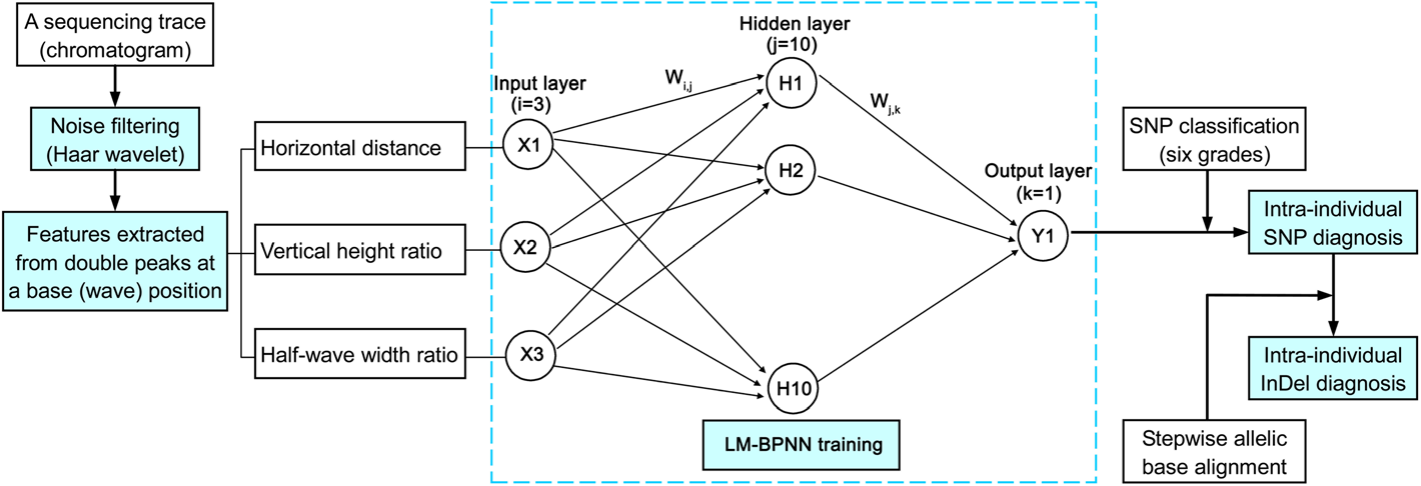
The overall structure of DiSNPindel. Haar wavelet transformation [11] was used to filter out the high-frequency noisy sub-signals of a base peak (wave). Three features were extracted from the primary and secondary peaks at a base position, namely, horizontal distance, vertical height ratio and half-wave width ratio, which were then inputted into LM-BPNN for intra-individual SNP diagnosis. The LM-BPNN contained three, ten and one neurons (vectors) in the input, hidden and output layers, respectively. Intra-individual InDel diagnosis was conducted using a stepwise allelic base alignment algorithm

DiSNPindel is a stand-alone package programmed in Matlab R2011b and LabWindows/CVI 9.0. It runs on Windows platform and can deal with multiple traces (.ab1 and/or .scf files), each being analyzed in an independent panel that is switchable between SNP and InDel interfaces. The method is composed mainly of four procedures, namely, noise filtering, feature extraction, SNP diagnosis and, if applicable, InDel diagnosis. Figure 1 summarizes the overall structure of DiSNPindel.

### Noise filtering

The Haar wavelet transformation [11] was used to decompose a peak (wave) signal into a low-frequency and a high-frequency sub-signals. While the high-frequency sub-signal would be removed as noises, the low-frequency sub-signal was subjected to further decomposition. The decomposition equation is:

$$ f(t)={A}_n+{\displaystyle \sum_{i=1}^n{D}_i} $$

where *f*(*t*) is the original signal, *A* is the approximation of low-frequency sub-signal (or further sub-signal), *D* is the details of high-frequency sub-signal (or further sub-signal) and *n* is the number of decomposition levels. More details of the Haar functions together with their parametric notations could be seen in literature, e.g., Stanković and Falkowski [13].

### Feature extraction

**Fig. 2:**
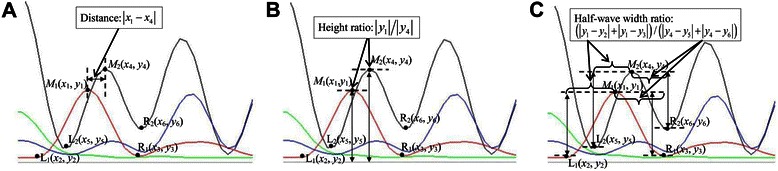
Three features extracted from double peaks (waves) at a base position. *L*, *M* and *R* indicate the positions of the left bottom, the middle top and the right bottom of a wave, respectively. **a** The horizontal distance (|*x*_1_ − *x*_4_|). **b** The vertical height ratio (|*y*_1_|/|*y*_4_|). **c** The half-wave width ratio [(|*y*_1_ − *y*_2_| + |*y*_1_ − *y*_3_|)/(|*y*_4_ − *y*_5_| + |*y*_4_ − *y*_6_|)]

For each chromatogram wave, horizontal distance, height and half-wave width were sampled from the primary (top) peak and, if applicable, the secondary (lower) peak, to represent the uniqueness of a wave position. Distance, height ratio and half-wave width ratio between the double peaks were then extracted as wave features efficient for subsequent diagnoses. Figure 2 shows the three features extracted from double peaks at a wave position.

### Intra-individual SNP diagnosis

Intra-individual SNPs were diagnosed with LM algorithm [14, 15] based BPNN [16, 17], which consisted of three layers: an input layer (the wave features), a hidden layer and an output layer (the score; Fig. 1). Using simulated within-individual SNP data, the LM-BPNN was trained for the output layer to meet a SNP score specification.

A total of 590 within-individual SNP samples synthetic of a wide range of the three wave features were simulated, including 443 and 147 for training and validation, respectively. An output value of each sample was generated using a fuzzy reasoning method [20, 21] and de-fuzzified to a score within the range 1–100.

LM-BPNN training was performed using the following weights and thresholds assumed for the three input, ten hidden and one output vectors (Fig. 1),

- The weight vector between the first input neuron and the ten neurons of the hidden layer: [*w*_(*1,1*)_^1^ *w*_(*1*,2)_^*1*^ … *w*_(1,1*0*)_^*1*^],
- The weight vector between the second input neuron and the ten neurons of the hidden layer: [*w*_(2,*1*)_^1^ *w*_(*2*,*2*)_^*1*^ … *w*_(*2*,*10*)_^*1*^],
- The weight vector between the third input neuron and the ten neurons of the hidden layer: [*w*_(*3*,1)_^1^ *w*_(*3*,*2*)_^*1*^ … *w*_(*3*,*10*)_^*1*^],
- The threshold vector of the hidden layer: [*b*_(1)_^1^ *b*_(*2*)_^*1*^ … *b*_(*10*)_^*1*^],
- The weight vector between the ten neurons of the hidden layer and the output layer: [*w*_(*1*,1)_^*2*^ *w*_(*2*,*1*)_^*2*^ … *w*_(*10*,*1*)_^*2*^], and
- The threshold of the output layer: [*b*_(1)_^2^].

In practice, the thresholds [*b*_(1)_^*1*^ *b*_(*2*)_^*1*^ … *b*_(*10*)_^*1*^] and [*b*_(1)_^2^] were treated as specific weights [*w*_(*0*,1)_^*1*^ *w*_(*0*,*2*)_^*1*^ … *w*_(*0*,*10*)_^*1*^] and [*w*_(*0*,*1*)_^2^], respectively. To determine the weight *w*_(*i*,*j*)_^*k*^ (*i* = 0, …, 3; *j* = 1, …, 10; *k* = 1, 2), the LM algorithm is used

$$ \varDelta w=-{\left[{J}^T(w)J(w)+\mu I\right]}^{-1}J(w)e(y) $$

where *w* is the weight vector, ∆*w* is the deviation of *w*, *J*(*w*) is the Jacobian matrix of vector *w*, *μ* is a coefficient, *I* is the identity matrix, and *e*(*y*) is the error. The learning steps are as follows.

1. Values between 0 and 1 were randomly assigned for initial weights and thresholds assuming a maximum error ε = 0.1.
2. Compute the BPNN output, e.g., ${y}_k={\displaystyle \sum_{i=1}^{10}{v}_i}{w}_i+{b}_{(1)}^2$ for the *k*th sample, where *v* is the input vector, *w* is the weight vector, and *b*_(1)_^2^ is the threshold of neuron *y*_l_. The error is thus calculated as *e*_*k*_ = *d*_*k*_ − *y*_*k*_, where *d*_*k*_ is the score calculated from the simulated data.
3. Compute the sum of square errors $V(w)={\displaystyle \sum_{i=1}^N{e}_k^2}$, where *N* is the number of samples.
4. If *V*(*w*) < *ε*, turn to step 7 below. Otherwise, compute the Jacobian matrix *J*(*w*).
5. Compute ∆*w* using the LM equation as stated above.
6. Let *w*(*t* + 1) = *w*(*t*) + *Δw* and compute the new sum of square errors *V*(*w*(*t* + 1)) similarly as in step 3 above. If *V*(*w*(*t* + 1)) < *V*(*w*(*t*)), set *V*(*w*(*t*)) = *V*(*w*(*t* + 1)), *w*(*t*) = *w*(*t* + 1) and *μ* = *μ*/*β* (*β* is a correction factor) and turn back to step 4; Otherwise, suppose *μ* = *μ* · *β* and turn back to step 5.
7. Reach the optimal weights and end the training process.

Finally a true SNP was trained to a score ≥75, a vague SNP to a score smaller than 75 but no less than 60, a false SNP to a score smaller than 60 but no less than 20, and a strongly false SNP to a score <20. In addition, using another set of 147 simulated samples, the performance of the trained LM-BPNN was tested.

**Fig. 3:**
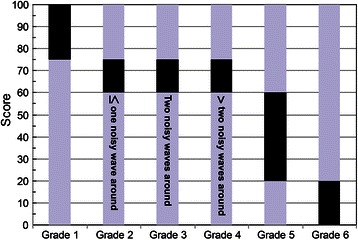
The six SNP grades classified based on the score and noisy peaks around. The score range for each grade was shadowed in black. Description of the number of noisy peaks around was stated on the column for grades 2–4

A six-grade classification was established for the convenience of SNP diagnosis (Fig. 3), that is, grade 1 with a score ≥ 75 (a true SNP), grades 2–4 being 60 ≤ score < 75 (a vague SNP), grade 5 being 20 ≤ score < 60 (a false SNP) and grade 6 with a score < 20 (a strongly false SNP). Grades 2–4 were further distinguished with the number of noisy waves (height and width more than 70 and 50 % of the secondary peak, respectively), that is, grades 2 (a true SNP), 3 (a possibly true SNP) and 4 (a possibly false SNP) with no more than one, two and more than two noisy waves around, respectively. Grades 1 and 2 could be a high threshold for SNP diagosis while grades 3 and 4 could be a relaxed threshold. Meanwhile, each SNP detected could be manually modified.

### Intra-individual InDel diagnosis

A stepwise allelic base alignment algorithm was employed for intra-individual InDel detection. A maximal InDel size was set at 30 bases according to Bhangale et al. [9]. The presumed primary and secondary peak sequences were compared for a given region dominated with continuous double peaks, supposing an interval of *m* (*m* = ±1, ±2, … or ±30) bases to reach the maximal matchability and allowing for base transposition between top and secondary peaks at a potentially misleading position. The final *m* value indicates an InDel of the *m* bases, and its signal + or – represents the presence of an insertion or deletion as compared to the alternative sequence.

### Datasets

As there was no ‘standard’ dataset tested with earlier detection tools, we benchmarked our software DiSNPindel with the three packages Mutation Surveyor, novoSNP and PolyPhred using a set of 62 *Eucalyptus* EST amplicons (Additional file 2) for SNP detection, which had been directly sequenced for wet-lab validation of a total of 66 SNPs associated with cleaved amplified polymorphic sequence (CAPS) markers in one or both parents of an F_1_ mapping population [22].

We also compared the performance of DiSNPindel with the four packages Mutation Surveyor, novoSNP, PolyPhred and PrimeIndel for InDel detection using 77 directly sequenced amplicons (Additional file 2) that contained intra-individual variation in simple sequence repeats (SSR) in either or both of the parents of an F_1_ mapping population [23].

## Results

### Noise filtering

**Fig. 4:**
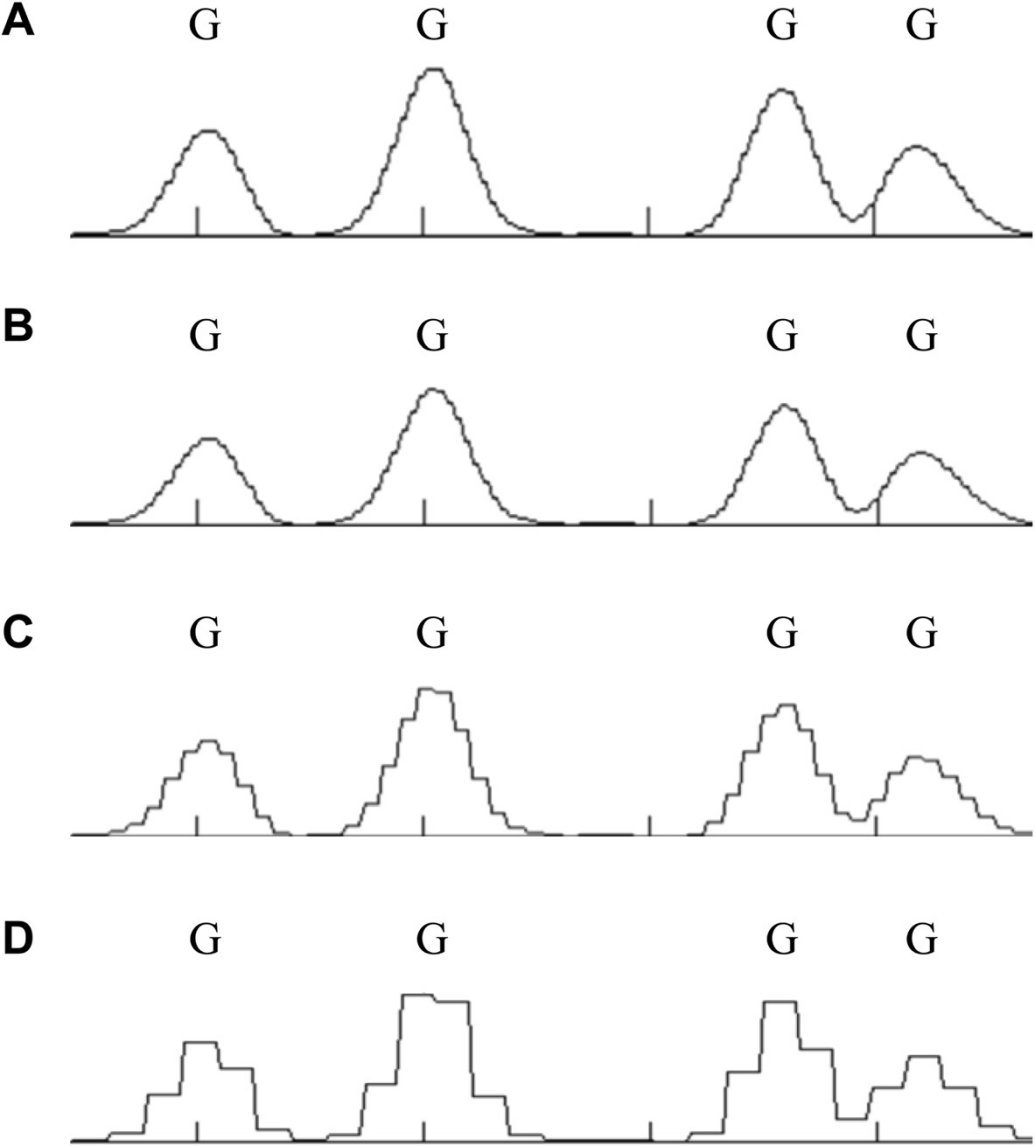
Approximation of the low-frequency sub-signal at three decomposition levels in the Haar wavelet transformation. **a** The original signal *f*(*t*). **b** The low-frequency sub-signal *A*_*1*_ generated at the first level of decomposition. **c** The low-frequency sub-signal *A*_*2*_ generated at the second level of decomposition. **d** The low-frequency sub-signal *A*_*3*_ generated at the third level of decomposition

After several rounds of trials, three levels of decomposition were finally applied, in which the high-frequency sub-signals at all levels were filtered out and the final low-frequency sub-signal (A3) was reserved to display the base position and the peak features. Figure 4 shows the low-frequency sub-signal at each level of the decomposition process.

### Intra-individual SNP diagnosis

**Fig. 5:**
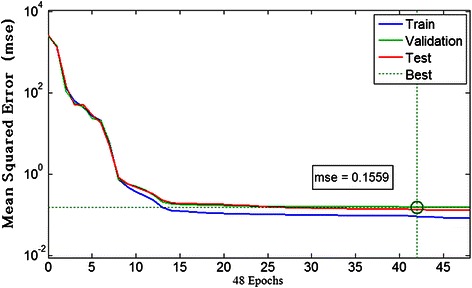
Performance in training, validation and test of the LM-BPNN. The mean squared error decreased rapidly to a stably low level in training, validation and test, indicating a strong convergence of all the three procedures. The best performance was reached at 42 interations in validation (MSE = 0.1559)

Totally 110 iterations were performed for LM-BPNN training. The mean squared error (MSE) decreased rapidly and reached a stably low level after about 13 iterations in the training and validation, indicating a strong convergence (Fig. 5). In particular, the best performance in validation was reached in 42 interations (MSE = 0.1559; Fig. 5). Similarly, MSE reached rapidly a stably low level in the test procedure (Fig. 5).

We compared our software with Mutation Surveyor, novoSNP and PolyPhred (v6.18) in intra-individual SNP diagnosis. As Polyphred was limited in analyzing a single trace, it did not detect any SNPs for all the ranks (1–6) and was excluded from subsequent comparisons. Of the total of 66 CAPS-related SNPs validated experimentally, our software showed the highest rate of found SNPs and the lowest rate of missed SNPs even at the highest threshold when compared to the relaxed thresholds of novoSNP and Mutation Surveyor. For instance, the found SNP rate was 90.9 % (60/66) at grade 1 in DiSNPindel, much higher than that of the most relaxed threshold in novoSNP (37.9 % at score 1) or Mutation Surveyor (30.3 % at high sensitivity; Table 1, Additional file 3: Table S1). Moreover, no SNP found by novoSNP and/or Mutation Surveyor was missed by DiSNPindel even at relatively higher grades. Furthermore, DiSNPindel enabled detection of four CAPS-SNPs subsequent to InDel (Additional file 1: Figure S3).

**Table 1:** Comparison of software performance in intra-individual SNP detection

Software^a^	Grade or score threshold^b^	Found SNPs (%)	Missed SNPs (%)
DiSNPindel	1	60 (90.9 %)	6 (9.1 %)
	2	61 (92.4 %)	5 (7.6 %)
	3	61 (92.4 %)	5 (7.6 %)
	4	63 (95.4 %)	3 (4.6 %)
	5	63 (95.4 %)	3 (4.6 %)
	6	63 (95.4 %)	3 (4.6 %)
novoSNP	18	0 (0.0 %)	66 (100.0 %)
	13	11 (16.7 %)	55 (83.3 %)
	9	16 (24.4 %)	50 (75.6 %)
	6	19 (28.8 %)	47 (71.2 %)
	3	25 (37.9 %)	41 (62.1 %)
	1	25 (37.9 %)	41 (62.1 %)
Mutation surveyor	Medium sensitivity	20 (30.3 %)	46 (69.7 %)
	High sensitivity	20 (30.3 %)	46 (69.7 %)

The numbers of found and missed SNPs were experimentally verified with 66 CAPS-associated intra-individual SNPs originating from 62 single traces [22]

^a^PolyPhred did not detect any SNPs at ranks 1–6 and was thus excluded from the comparison

^b^Grade 2, score 13 and medium sensitivity could be a high threshold for reliable SNP detection in DiSNPindel, novoSNP and Mutation Surveyor, respectively, where Grade 4, score 6 and high sensitivity could be a low threshold

**Fig. 6:**
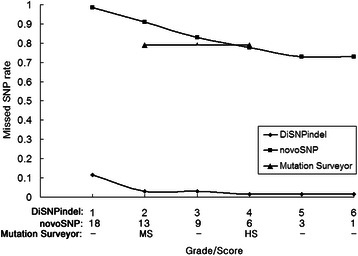
Missed SNP rates for 50 relatively high-quality single traces [22] by DiSNPindel, novoSNP and Mutation Surveyor at different grade or score thresholds. PolyPhred missed all the SNPs at all the ranks (1–6) and was excluded from the comparison. More data were given in Additional file 3: Table S2. MS: medium sensitivity; HS: high sensitivity

**Fig. 7:**
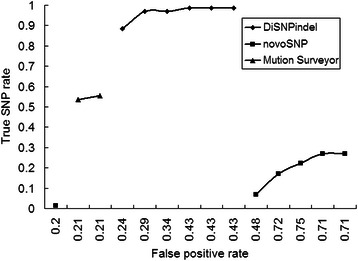
True SNP versus false positive SNP rates investigated for 50 relatively high-quality single traces [22] by DiSNPindel, novoSNP and Mutation Surveyor. PolyPhred missed all the SNPs at all the threshold ranks (1–6) and was excluded from the comparison. More data were given in Additional file 3: Table S2

Also, DiSNPindel outperformed novoSNP and Mutation Surveyor in efficacy of detection on all the SNPs identified manually from 50 relatively high-quality traces out of the 62 amplicons. DiSNPindel showed the lowest rate of missed SNPs at all the thresholds (Fig. 6, Additional file 3: Table S2) and the highest rate of true SNPs at a relatively low rate of false positives (Fig. 7, Additional file 3: Table S2).

### Intra-individual InDel diagnosis

As compared with Mutation Surveyor, novoSNP, PolyPhred (v6.18) and PrimeIndel, except Polyphred that could not detect any InDels, DiSNPindel resulted in the highest true InDel rate (53.1 %) but the least missed (22.2 %) and false positive (0 %) rates, plus the highest rate (24.7 %) of size-correct but base-wrong InDels (Table 2, Additional file 3: Table S3). Only one true InDel (trace eSSR509P2F; Additional file 3: Table S3) detected by Mutation Surveyor was missed by DiSNPindel and three true InDels (traces eSSR348P1F, eSSR479P1F and eSSR650P1F; Additional file 3: Table S3) detected by novoSNP and/or Mutation Surveyor were determined with correct size but wrong bases in our software.

**Table 2:** Comparison of software performance in intra-individual InDel detection

Software^a^	True InDels (%)	Size-correct InDels with wrong bases (%)	Missed InDels (%)	False positive InDels (%)
DiSNPindel	43(53.1 %)	20 (24.7 %)	18 (22.2 %)	0 (0.0 %)
novoSNP	6 (7.4 %)	7 (8.6 %)	68 (84.0 %)	34 (72.3 %)
Mutation surveyor	7 (8.6 %)	7 (8.6 %)	67 (82.8 %)	26 (65.0 %)
PrimeIndel	15 (18.5 %)	10 (12.3 %)	56 (69.1 %)	0 (0.0 %)

The numbers of true, size-correct but base-wrong, missed and false positive InDels were detected with 77 single traces containing SSR-associated InDels [23]. More data were given in Additional file 3: Table S3

^a^PolyPhred did not detect any InDels and was thus excluded from the comparison

## Discussion

Resequencing based on the Sanger method has been the gold standard for discovery of DNA polymorphisms in a specific genomic region, given that the relatively high error rates in NGS reads will cause inevitably false SNPs [24, 25]. In case of SNP and InDel discovery within an individual (heterozygote), attentions are mostly paid to a single sequence trace file of PCR amplicon rather than multiple traces with additional individuals. In this regard, our method focusing on a single trace irrespective of reference sequence represents a considerable advance towards automated within-individual SNP and InDel identification in a diploid.

As shown herein, our software outperformed other contemporary methods in accuracy of intra-individual SNP and InDel detection. The better performance can be largely attributable to the effective algorithm for SNP diagnosis and, accordingly, the accurate allelic comparison for InDel determination. To our knowledge, wavelet transformation and BPNN are for the first time introduced for studies of the kind. Besides eliminating the need for reference sequence, the significantly improved accuracy in both SNP and InDel detection by DiSNPindel indicates that our novel algorithm provides a reliable and efficient alternative for automated detection of sequence variations. In addition, DiSNPindel could result in consistent diagnoses from different runs of the same sequencing trace.

Though DiSNPindel is designed to detect new SNPs and InDels within an individual, it can be used to genotype multiple samples, with each output saved in a txt file. Moreover, the output sequences could be aligned for multiple sample comparison using a third-part program, e.g., Clustal W [26].

Based on the setting of six confidence grades in SNP detection, DiSNPindel allows the choice of threshold to distinguish between true and false positives, thereby enabling a tradeoff between missed and erroneous SNPs. For instance, the true, false positive and missed SNP rates were 97.0, 29.2 and 3.0 %, respectively, at a high threshold of grade 2, but were 98.5, 42.9 and 1.5 %, respectively, at a lower threshold of grade 4 (Figs. 6 and 7, Additional file 3: Table S2). A proper threshold value may depend on practical application [6, 8]. Nevertheless, the relatively high false positive rate even at a high threshold suggests the necessity of manual review, especially for certain circumstances such as mutation detection and clinical diagnosis [6]. Moreover, as the false positive rate is directly correlated with the sequence trace quality [8], optimization of PCR condition and sequencing primer could be helpful to reduce the false positive rate.

## Conclusions

In this report, a novel yet efficient tool was proposed for intra-individual SNP and InDel detection in diploid amplicon sequencing. It will also be useful for identification of DNA variation in EST re-sequencing. The proposed tool does not require a reference sequence or additional samples. Moreover, as compared with existing detection methods, it can improve largely the true SNP and InDel discovery rates and reduce largely the missed and false positive rates. In addition, the tool can be used to genotype multiple samples.

## Availability and requirements

DiSNPindel (as of version 1.0) is freely available to all readers at http://www.ritf.ac.cn/sitecn/FZBJKYCG/1377.html.

**Project name:** DiSNPindel

**Project home page:** http://www.ritf.ac.cn/sitecn/FZBJKYCG/1377.html

**Operating system(s):** Windows XP or higher

**Programming language:** Matlab

**License:** none

**Any restrictions to use by non-academics:** none

## Additional files

Additional file 1: Figure S1. A SNP detection interface in software DiSNPindel (http://www.ncbi.nlm.nih.gov/nucest/CB967984). **Figure S2.** An InDel detection interface in software DiSNPindel. **Figure S3.** CAPS-SNPs subsequent to InDel could be identified by software DiSNPindel. (PDF 266 kb)
Additional file 2:
**Test Data.** (ZIP 9355 kb)
Additional file 3: Table S1. Found and missed SNPs out of the 66 CAPS-associated intra-individual SNPs from 62 single traces [22] by four software packages DiSNPindel, novoSNP [8], Mutation Surveyor (http://www.softgenetics.com/mutationSurveyor.html) and PolyPhred [6, 9]. **Table S2.** True, false positive and missed SNPs by four software packages DiSNPindel, novoSNP [8], Mutation Surveyor (http://www.softgenetics.com/mutationSurveyor.html) and PolyPhred [6, 9] for 50 amplicons previously directly sequenced [22]. Those sequencing traces with no detection results shown in novoSNP and/or unmatched with reference sequence in Mutation Surveyor were excluded. **Table S3.** InDels detected by four software packages DiSNPindel, novoSNP [8], Mutation Surveyor (http://www.softgenetics.com/mutationSurveyor.html), PolyPhred [6, 9] and PrimeIndel [10] for 77 previously directly sequenced amplicons that contained within-individual variation in simple sequence repeats [23]. (XLS 399 kb)

## Abbreviations

SNP: Single nucleotide polymorphisms
InDel: Insertion-deletion
PCR: Polymerase chain reaction
EST: Expressed sequence tag
NGS: Next-generation sequencing
LM: Levenberg-Marquardt
BPNN: Back-propagation neural networks
CAPS: Cleaved amplified polymorphic sequence
SSR: Simple sequence repeats
MSE: Mmean squared error
