# Supplementary material for: DiSNPindel: improved intra-individual SNP and InDel detection in direct amplicon sequencing of a diploid
Source: BMC Bioinformatics. 2015 Oct 24;16:343. doi: 10.1186/s12859-015-0790-y (PMC4619477; doi:10.1186/s12859-015-0790-y)
Supplement: Additional file 1: Figure S1. — A SNP detection interface in software DiSNPindel (http://www.ncbi.nlm.nih.gov/nucest/CB967984). Figure S2. An InDel detection interface in software DiSNPindel. Figure S3. CAPS-SNPs subsequent to InDel could be identified by software DiSNPindel. (PDF 266 kb) [file 12859_2015_790_MOESM1_ESM.pdf]

# Additional file 1

## DiSNPindel: improved intra-individual SNP and InDel detection in direct amplicon sequencing of a diploid

Jizhong Deng, Huasheng Huang, Xiaoli Yu, Ji Jin, Weisen Lin, Fagen Li, Zhijiao Song, Mei Li and Siming Gan

## Figures S1–S3

**Figure S1 A SNP detection interface in software DiSNPindel.** Each of the sequential steps corresponds to a button or an operational box. The amplicon for direct sequencing shown above was generated with the male *Eucalyptus tereticornis* parent ($P_2$) of an *E. urophylla* × *E. tereticornis* $F_1$ mapping population [22] against the target EST accession CB967984 (http://www.ncbi.nlm.nih.gov/dbEST/). Two intra-individual SNPs were shown here at 182 and 190 bp assigned with scores 87 and 72 as well as Grades 1 and 2, respectively.

**Figure S2 An InDel detection interface in software DiSNPindel.** Each of the sequential steps corresponds to a button or an operational box. The amplicon for direct sequencing shown above was generated with the female *Eucalyptus urophylla* parent ($P_1$) of an *E. urophylla* × *E. tereticornis* $F_1$ mapping population [23] against the target EST accession ES596705 (http://www.ncbi.nlm.nih.gov/dbEST/). A 12-bp insertion of $(CTCC)_2$CTCT was shown in the secondary-peak sequence (101–112 bases) with an accuracy of 0.98.

**Figure S3 CAPS-SNPs subsequent to InDel could be identified by software DiSNPindel.** These CAPS-SNPs were verified experimentally in previous work [22]. (**A**) TTTA[T/A]A (circled in blue) corresponding to *Dra*I digestion site TTT*AAA was subsequent to a 2-bp InDel for marker *EUCeC*11 (GenBank EST accession CD669354) in *Eucalyptus tereticornis* parent ($P_2$) of an *E. urophylla* × *E. tereticornis* $F_1$ mapping population [22]. (**B**) C[A/C]TGG (circled in blue) corresponding to *Mva*I digestion site CC*(A/T)GG was subsequent to a 6-bp InDel for marker *EUCeC*12 (GenBank EST accession CD669252) in $P_2$ of the $F_1$ mapping population [22]. (**C**) C[C/G]T[GG/TT] (circled in blue) corresponding to *Mva*I digestion site CC*(A/T)GG was subsequent to a 13-bp InDel for marker *EUCeC*43 (GenBank EST accession CD670044) in $P_2$ of the $F_1$ mapping population [22]. (**D**) CCT[A/G]G (circled in blue) corresponding to *Mva*I digestion site CC*(A/T)GG was subsequent to a 5-bp InDel for marker *EUCeC*63 (GenBank EST accession CD668142) in *Eucalyptus urophylla* parent ($P_1$) of the $F_1$ mapping population [22].

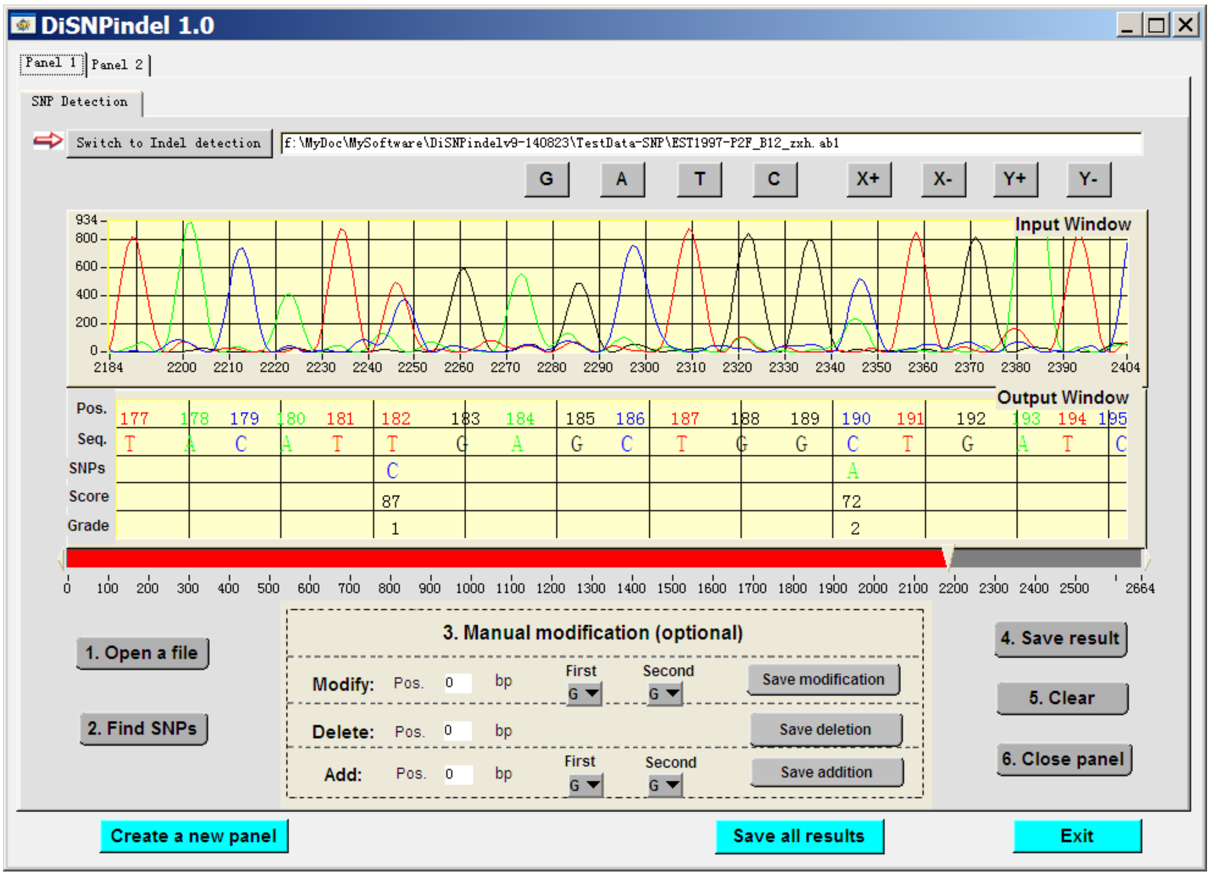


**Figure S1 A SNP detection interface in software DiSNPindel.** Each of the sequential steps corresponds to a button or an operational box. The amplicon for direct sequencing shown above was generated with the male *Eucalyptus tereticornis* parent ($P_2$) of an *E. urophylla* × *E. tereticornis* $F_1$ mapping population [22] against the target EST accession CB967984 (http://www.ncbi.nlm.nih.gov/dbEST/). Two intra-individual SNPs were shown here at 182 and 190 bp assigned with scores 87 and 72 as well as Grades 1 and 2, respectively.

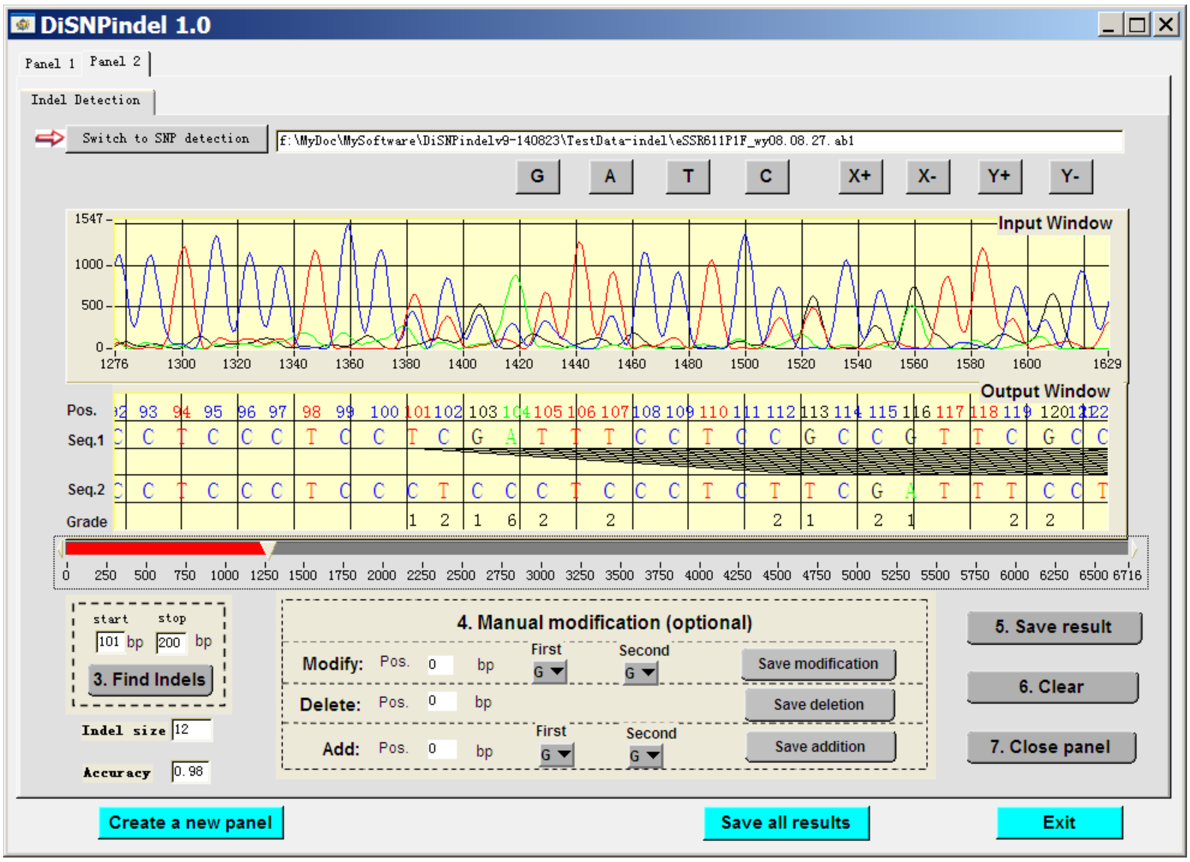


**Figure S2 An InDel detection interface in software DiSNPindel.** Each of the sequential steps corresponds to a button or an operational box. The amplicon for direct sequencing shown above was generated with the female *Eucalyptus urophylla* parent ($P_1$) of an *E. urophylla* × *E. tereticornis* $F_1$ mapping population [23] against the target EST accession ES596705 (http://www.ncbi.nlm.nih.gov/dbEST/). A 12-bp insertion of $(CTCC)_2$CTCT was shown in the secondary-peak sequence (101–112 bases) with an accuracy of 0.98.

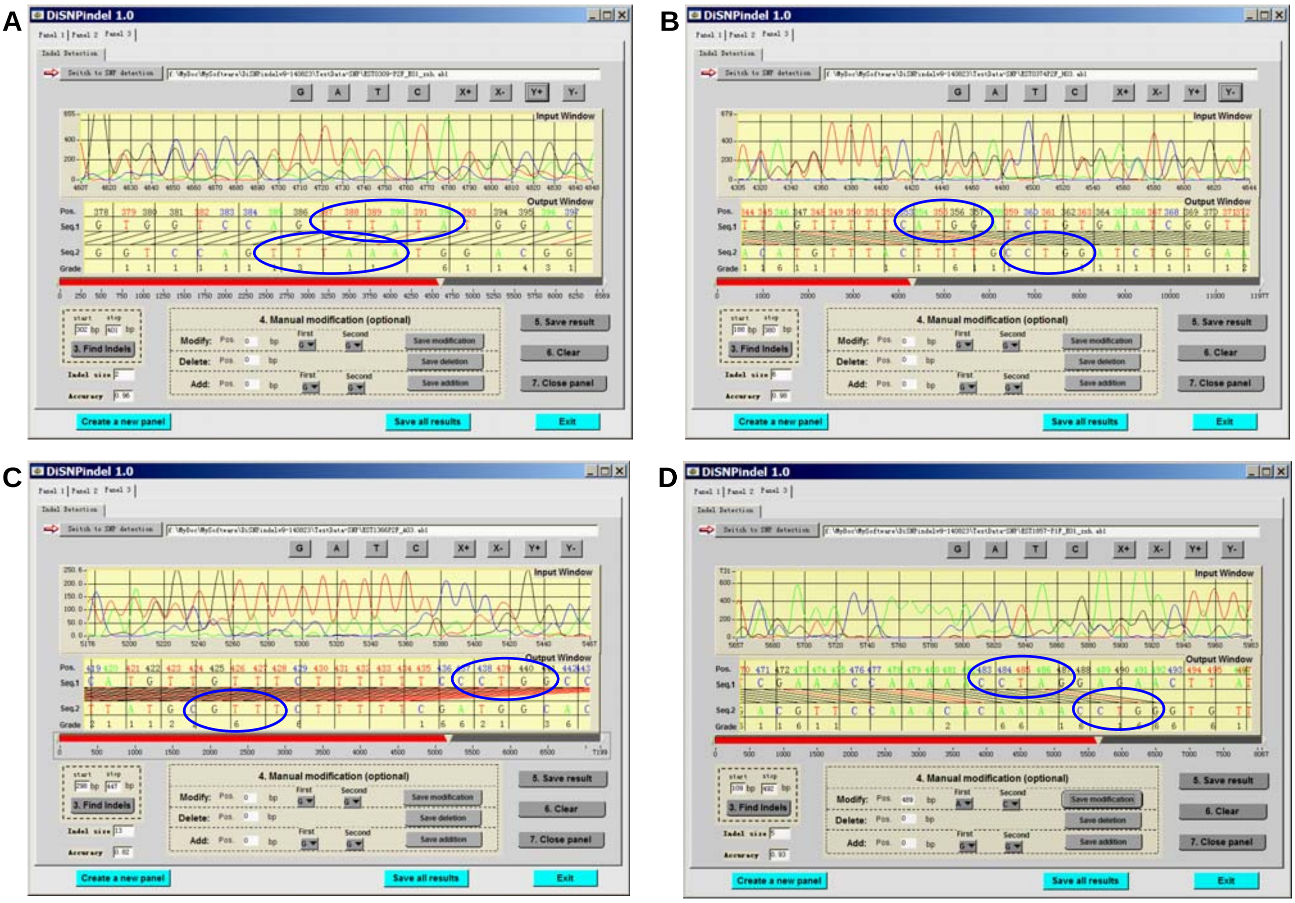

**Figure S3 CAPS-SNPs subsequent to InDel could be identified by software DiSNPindel.** These CAPS-SNPs were verified experimentally in previous work [22]. (**A**) TTTA[T/A]A (circled in blue) corresponding to *Dra*I digestion site TTT*AAA was subsequent to a 2-bp InDel for marker *EUCeC*11 (GenBank EST accession CD669354) in *Eucalyptus tereticornis* parent ($P_2$) of an *E. urophylla* × *E. tereticornis* $F_1$ mapping population [22]. (**B**) C[A/C]TGG (circled in blue) corresponding to *Mva*I digestion site CC*(A/T)GG was subsequent to a 6-bp InDel for marker *EUCeC*12 (GenBank EST accession CD669252) in $P_2$ of the $F_1$ mapping population [22]. (**C**) C[C/G]T[GG/TT] (circled in blue) corresponding to *Mva*I digestion site CC*(A/T)GG was subsequent to a 13-bp InDel for marker *EUCeC*43 (GenBank EST accession CD670044) in $P_2$ of the $F_1$ mapping population [22]. (**D**) CCT[A/G]G (circled in blue) corresponding to *Mva*I digestion site CC*(A/T)GG was subsequent to a 5-bp InDel for marker *EUCeC*63 (GenBank EST accession CD668142) in *Eucalyptus urophylla* parent ($P_1$) of the $F_1$ mapping population [22].

## References

22. Yu X, Guo Y, Zhang X, Li F, Weng Q, Li M, Gan S: Integration of EST-CAPS markers into genetic maps of *Eucalyptus urophylla* and *E. tereticornis* and their alignment with *E. grandis* genome sequence. Silvae Genet. 2012;61:247–55.

23. He X, Wang Y, Li F, Weng Q, Li M, Xu L, Shi J, Gan S: Development of 198 novel EST-derived microsatellites in *Eucalyptus* (Myrtaceae). Am J Bot. 2012;99:e134–48.
